# A dataset on the physiological state and behavior of drivers in conditionally automated driving

**DOI:** 10.1016/j.dib.2023.109027

**Published:** 2023-03-03

**Authors:** Quentin Meteier, Marine Capallera, Emmanuel de Salis, Leonardo Angelini, Stefano Carrino, Marino Widmer, Omar Abou Khaled, Elena Mugellini, Andreas Sonderegger

**Affiliations:** a HumanTech Institute, University of Applied Sciences and Arts of Western Switzerland, HES-SO, Boulevard de Pérolles 80, Fribourg, 1700, Switzerland; b Haute-Ecole Arc Ingénierie, University of Applied Sciences and Arts of Western Switzerland, HES-SO, Rue de la Serre 7, Saint-Imier, 2610, Switzerland; c School of Management Fribourg, University of Applied Sciences and Arts of Western Switzerland, HES-SO, Chemin du Musée 4, Fribourg, 1700, Switzerland; d University of Fribourg, Department of Informatics, Boulevard de Pérolles 90, Fribourg, 1700, Switzerland; e Bern University of Applied Sciences, Business School, Institute for New Work, Brückenstrasse 73, Bern, 3005, Switzerland

**Keywords:** Conditionally automated driving, Driver state, Physiology, Electrocardiogram (ECG), Electrodermal activity (EDA), Respiration, Situation awareness (SA), Takeover quality

## Abstract

This dataset contains data of 346 drivers collected during six experiments conducted in a fixed-base driving simulator. Five studies simulated conditionally automated driving (L3-SAE), and the other one simulated manual driving (L0-SAE). The dataset includes physiological data (electrocardiogram (ECG), electrodermal activity (EDA), and respiration (RESP)), driving and behavioral data (reaction time, steering wheel angle, …), performance data of non-driving-related tasks, and questionnaire responses. Among them, measures from standardized questionnaires were collected, either to control the experimental manipulation of the driver's state, or to measure constructs related to human factors and driving safety (drowsiness, mental workload, affective state, situation awareness, situational trust, user experience).

In the provided dataset, some raw data have been processed, notably physiological data from which physiological indicators (or features) have been calculated. The latter can be used as input for machine learning models to predict various states (sleep deprivation, high mental workload, ...) that may be critical for driver safety. Subjective self-reported measures can also be used as ground truth to apply regression techniques. Besides that, statistical analyses can be performed using the dataset, in particular to analyze the situational awareness or the takeover quality of drivers, in different states and different driving scenarios.

Overall, this dataset contributes to better understanding and consideration of the driver's state and behavior in conditionally automated driving. In addition, this dataset stimulates and inspires research in the fields of physiological/affective computing and human factors in transportation, and allows companies from the automotive industry to better design adapted human-vehicle interfaces for safe use of automated vehicles on the roads.

**Specifications Table**

**Table:** 

Subject	*List of DIB categories not available (link not found)*
Specific subject area	Physiology Work psychology and cognitive ergonomics Computer science
Type of data	Physiological data Driving data Socio-demographic data Subjective measures Performance measures
How the data were acquired	Fixed-base driving simulator with one or two seats, a pedal set, a Logitech G27 or G29 steering wheel and the driving scenario displayed whether on a large screen with a projector or a television screen (65”). Physiological data: - Biopac MP36 with lead sets and electrodes, collected with Biopac Student lab 3.7.7. - In the final experiment, collected with Biosignalsplux hardware. Driving data acquired from open source driving simulation softwares: OpenDS and GENIVI. Performance measures: Samsung Galaxy Tab A and GENIVI software. Demographic and questionnaire data: Unipark^1^ The experimental procedure, design, material and instruments are detailed in a README file in each folder of the data repository.
Data format	Raw Aggregated Filtered and processed
Description of data collection	It is a dataset gathering data from 346 drivers, collected in 6 fixed-base driving simulator experiments. Five of them simulated conditionally automated driving (L3-SAE) and one simulated manual driving (L0-SAE). Each folder contains raw and preprocessed data collected in each experiment. It contains three physiological signals (electrocardiogram (ECG), electrodermal activity (EDA),
	and respiration (RESP)), driving data, socio-demographic data, and self-reported ratings on standardized scales and questionnaires*.*
Data source location	*·* Institutions: (1) University of Fribourg (2) University of Applied Sciences and Arts of Western Switzerland (HES-SO)
	· City: Fribourg · Country: Switzerland · Latitude and longitude (and GPS coordinates, if possible) for collected samples/data: (1) 46.79661602839843, 7.1565483249698305 (2) 46.793461030370345, 7.159055598178482
Data accessibility	Repository name: Zenodo Data identification number: 10.5281/zenodo.7214953 Direct URL to data: https://doi.org/10.5281/zenodo.7214953

^1^https://www.unipark.com/.

## Value of the Data

• This dataset [1] gathers heterogeneous data (driving, physiological, behavioral, performance, questionnaire responses) collected from a large number (N = 346) of individual drivers in different psychophysiological states (fatigue, mental workload, affective state), specifically in the context of conditionally automated driving (L3-SAE). To date, such a dataset does not exist.
• Further quantitative analyses (in addition of those made in the referenced publications) can be conducted using the large range of measure collected in different situations of conditionally automated driving. This can help to better understand the role of human factors and driving situation in such context, helping to define guidelines for the design of human-vehicle interfaces, to support drivers and increase safety on roads.
• In the field of affective and physiological computing, several research questions can be investigated on the basis of this dataset, such as determining the most predictive physiological indicators of certain psychophysiological states (stress, mental workload or fatigue), along with the optimal time windows for assessing them. The consideration of a baseline (i.e., the physiological state at rest) for assessing someone's condition can also be investigated.
• In the field of computer science, the physiological dataset can serve in developing innovative artificial intelligence models to assess the driver's state, including the consideration of several psychophysiological states such as mental workload, fatigue, or the affective state. Such models could provide driver's biofeedback, and thus give the car the possibility (or not) to give back control to the driver according to his/her state.
• Automotive industries can also use the data to understand the driver's state and behavior in a simulated environment (in a research context). This can be a basis for designing human-vehicle interfaces implemented in future vehicles that will drive at this level of automation (L3-SAE) on roads.

## 1 Objective

The idea behind the creation of this dataset is the design of an adaptive autonomous system called AdVitam (for Advanced Driver-Vehicle Interaction to Make future driving safer). The goal of this system is to maintain the driver's situation awareness and takeover quality in conditionally automated driving (L3-SAE). To fulfill that role, the idea is to adapt dynamically the human-vehicle interaction, depending on the driver's state and the driving situation.

In order to develop this system and particularly the module assessing the driver's state, it was necessary to collect physiological data (electrocardiogram (ECG), electrodermal activity (EDA), and respiration (RESP)) from drivers in different states. Thus, several experiments were conducted on a fixed-base simulator. The collected data were used to train various machine learning models capable of predicting certain psychophysiological states (fatigue, mental workload, affective state) continuously. Both objective and subjective measures related to human factors linked with driving safety were also collected (takeover quality, situation awareness, trust, task performance, user experience). Based on statistical analyses, dynamically adaptable human-vehicle interfaces for supervision (lights on the dashboard, a haptic seat and a mobile application) and intervention (adaptive takeover modality) were designed. The overall AdVitam system was finally tested and evaluated in a preliminary user study with 35 drivers. The dataset contains all data collected in the framework of the AdVitam project.

## 2 Data Description

### 2.1 Global folder structure of the dataset

The dataset [1] consists of data collected in 6 different experiments conducted on a fixed-base driving simulator. Each experiment is identified by a code: Exp1, Exp2, Exp3, Exp4, ExpTOR, ExpFinal. The purpose of each experiment is explained below:

- Exp1: Experimental manipulation of relaxation before driving and presence of passenger while driving (manual driving, L0-SAE)
- Exp2: Experimental manipulation of cognitive workload at 2 levels using a verbal task (backwards counting)
- Exp3: Experimental manipulation of cognitive workload at 3 levels using visual and auditory tasks (N-back task)
- Exp4: Experimental manipulation of fatigue (sleep deprivation) and driving environment (rural vs. urban scenario)
- ExpTOR: Multiple takeovers requested through different modalities (visual, auditory, haptic), while performing different non-driving related tasks
- ExpFinal: Testing a contextual multimodal system for maintaining situation awareness and takeover quality in conditionally automated driving

**Fig. 1:**
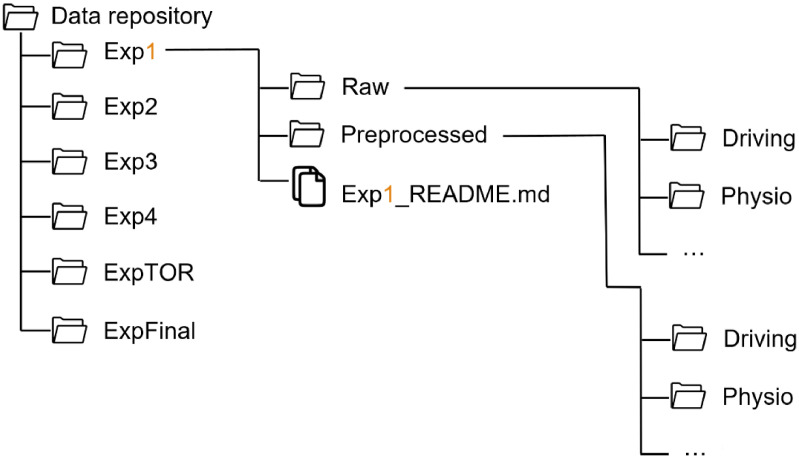
Global view of the folder structure

**Fig. 2:**
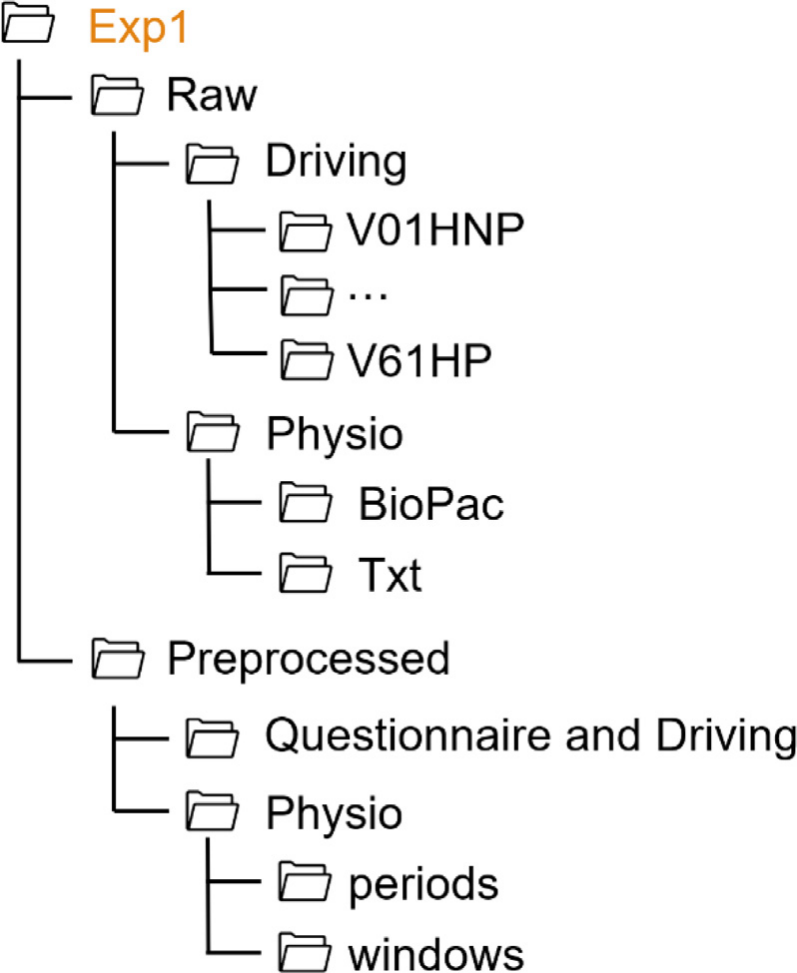
Folder structure of the Exp1 folder

**Fig. 3:**
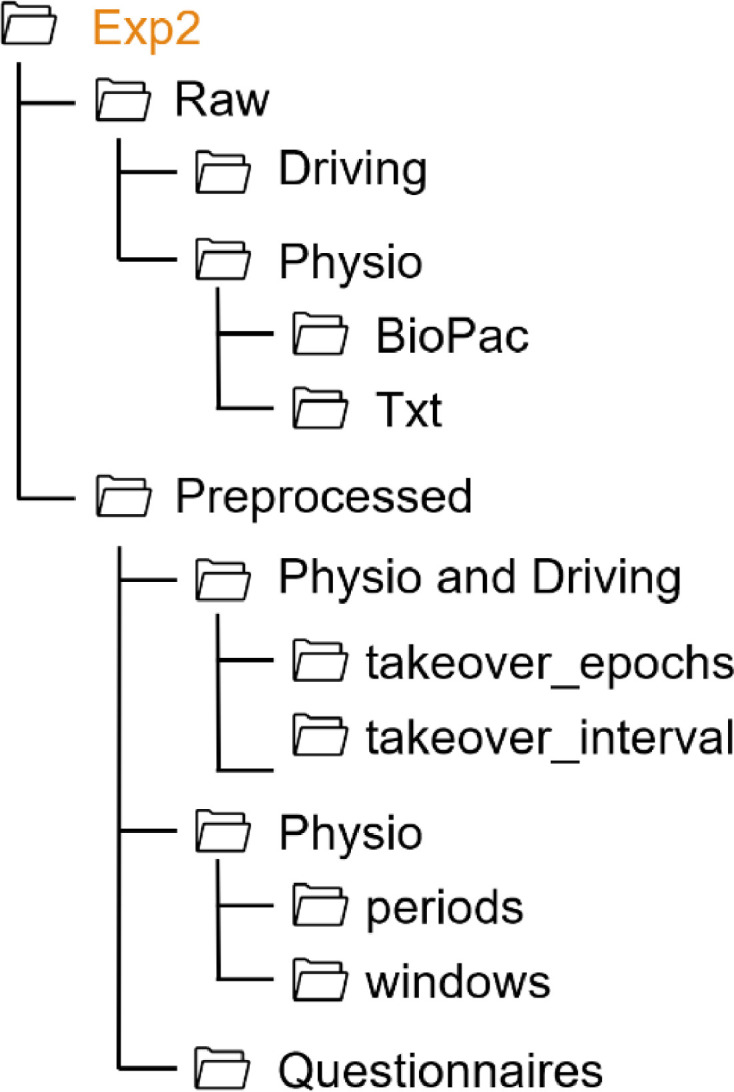
Folder structure of the Exp2 folder

**Fig. 4:**
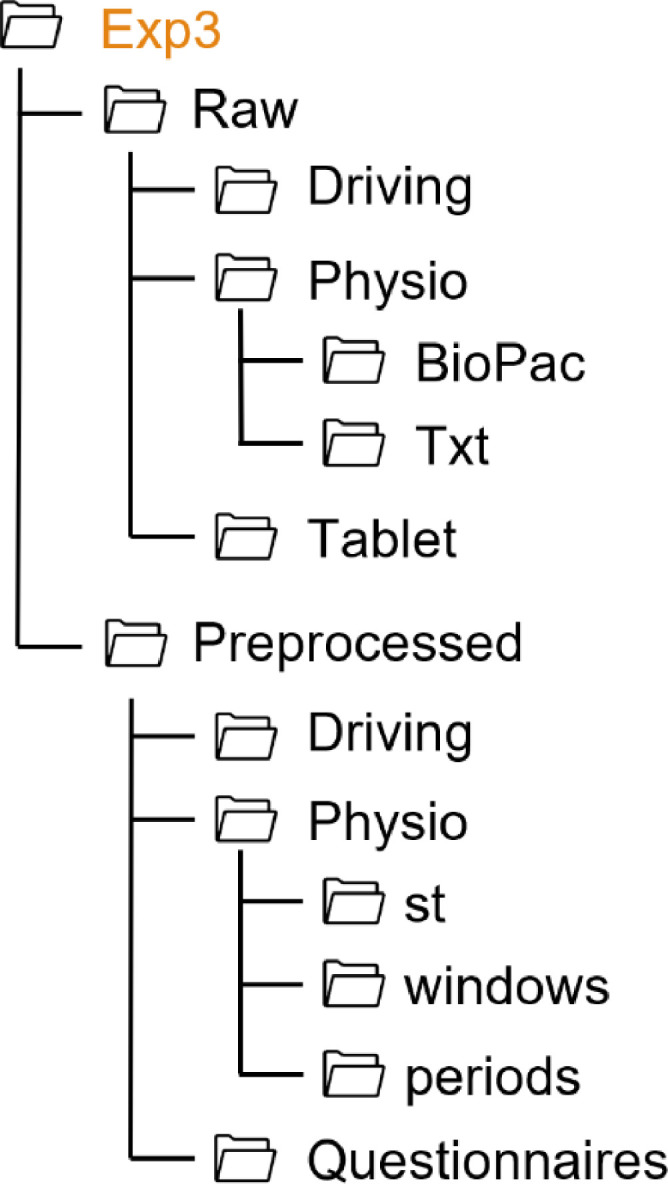
Folder structure of the Exp3 folder

**Fig. 5:**
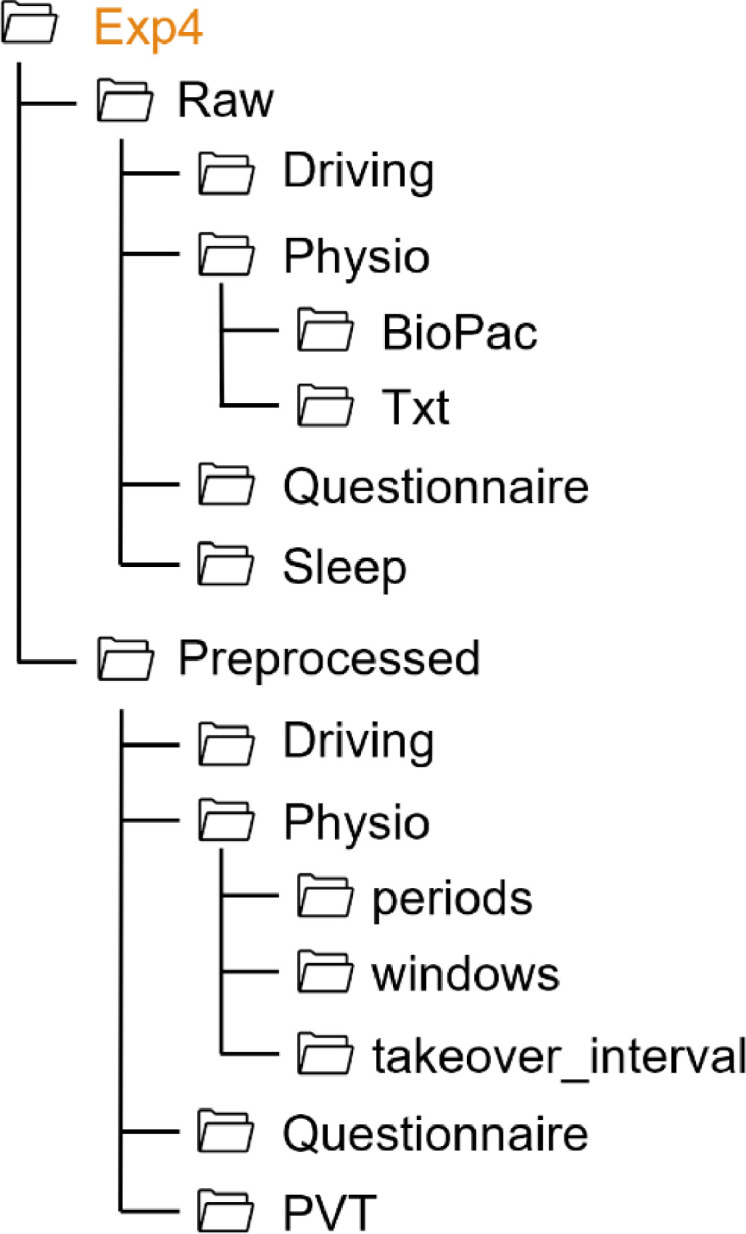
Folder structure of the Exp4 folder

**Fig. 6:**
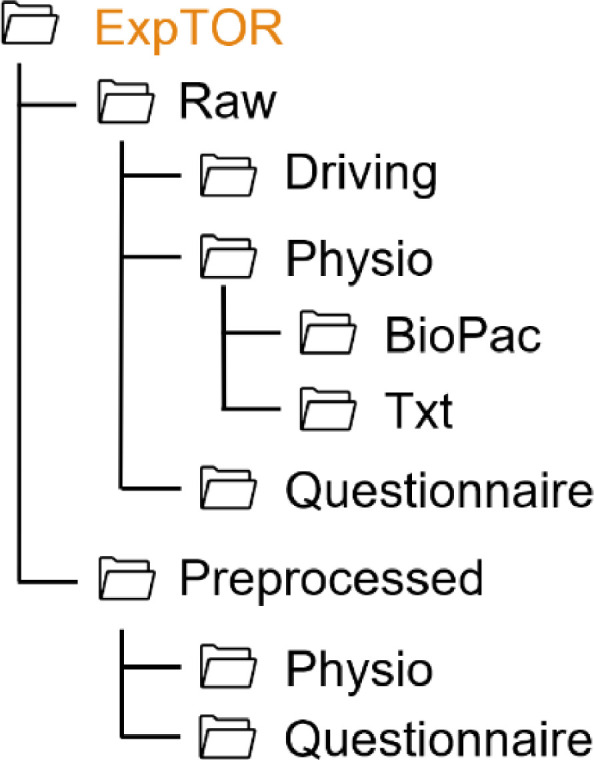
Folder structure of the ExpTOR folder

**Fig. 7:**
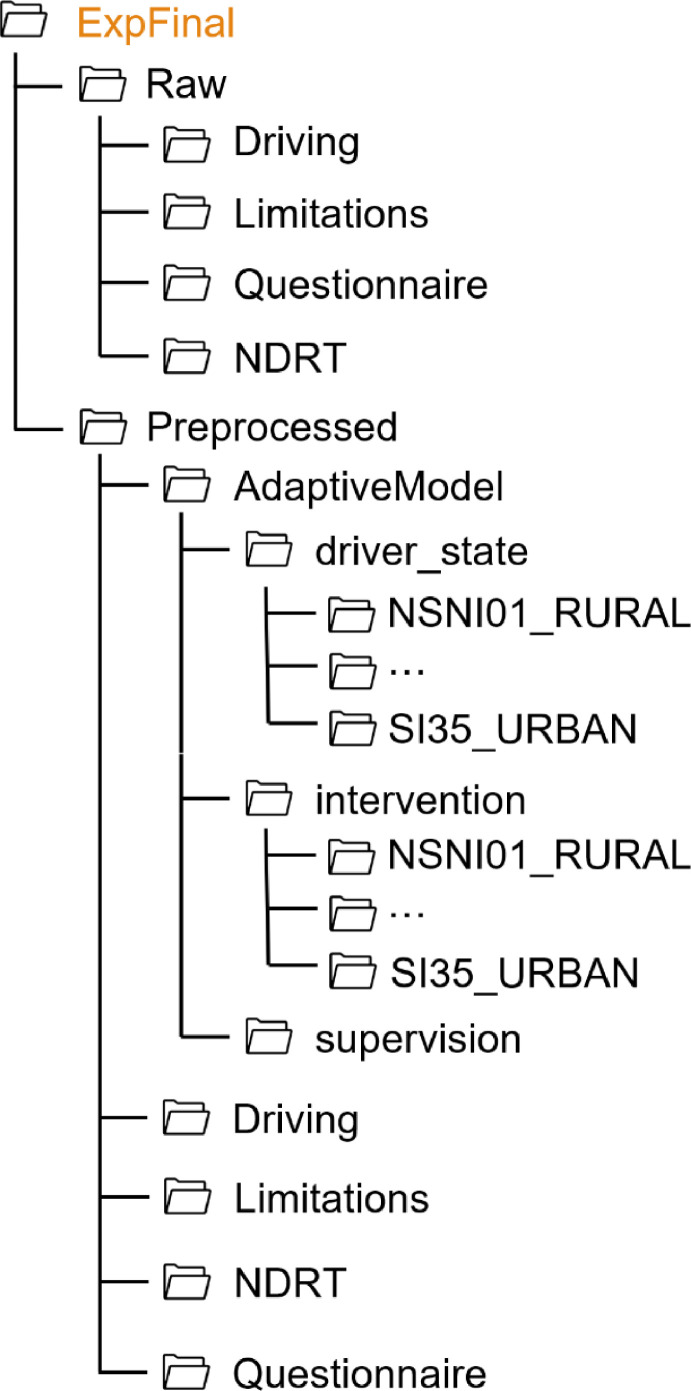
Folder structure of the ExpFinal folder

For all experiments, the folder structure follows the same pattern, as shown in Fig. 1. Each experiment folder contains two subfolders (Raw, Preprocessed) and a README file. The data collected in each experiment are stored in the respective folder. Since in each experiment additional measures were collected in addition to the standard data, the folder structure varies from one experiment to another. Thus, the structure of each experiment folder is shown on Fig. 2, Fig. 3, Fig. 4, Fig. 5, Fig. 6, Fig. 7.

### 2.2 Folder structure and metadata common to all experiments

#### 2.2.1 README file

The README file of each experiment contains an abstract of the experiment, a summary of the methods and material employed to conduct the study. It also contains information on the structure of the files and their content. Metadata (variables and coding) are also documented so that anyone can use every file contained in the folders. Relevant scientific references are also included.

#### 2.2.2 Raw data

The raw data are contained in the Raw folder. For all experiments, it contains driving data of each participant in a .txt format, contained in the Driving folder. For experiments that consisted of several scenarios/phases, there are several .txt files for one participant. Besides, all experiment folders (except ExpFinal) contain physiological raw data of drivers contained in a Physio folder. The data are available in .acq format (Biopac folder, raw files generated by the data collection software) and .txt format (Txt folder). In the Txt folder, there are two files associated with each participant, one file containing the raw data (ECG, EDA and RESP) and one file with timestamps corresponding to the beginning and the end of each experimental phase. For some experiments, there are other folders with other types of raw data: socio-demographic data and questionnaire data extracted from the online platform Unipark, or data collected from a mobile application developed specifically for the experiment and running on a handheld tablet.

• /Physio: contains two folders with physiological data collected during the experiment
  ○ /BioPac: contains the raw files (in .acq format) with physiological signals: ECG, EDA and RESP. These files were generated by the BioPac Student Lab 3.7.7 software, using the BioPac MP36 hardware for signal collection.
  ○ /Txt: contains two .txt files for each driver, identified with the code.
    ■ <code>.txt: contains the raw physiological data extracted from the BioPac Student Lab. Each column contains the raw values collected with sensors for each signal (ECG, EDA. RESP) at a sampling rate of 1000Hz. The file contains metadata in the first 11 rows. Columns are separated with tabs. The first column is the elapsed time in minutes.
    ■ <code>-markers.txt: contains the timestamps for each period of the experiment. Metadata corresponding to the timestamps in each experiment can be found in the README of each experiment. **Be careful, the timestamps are here in seconds while they are in minutes in the raw data** (<code>.txt)**.**
• /Driving: contains raw driving data collected from the driving simulation software (either OpenDS or GENIVI, see README of each experiment).
  ○ Metadata:
    ■ Time = Time elapsed since the software was launched (in seconds)
    ■ EngineSpeed = Engine speed (in rpm)
    ■ GearPosActual = Current gear
    ■ GearPosTarget = Next planned gear
    ■ AcceleratorBrakePedalPos = Position of gas/brake pedal. Gas pedal is pressed when the value is between 0 and 1 (maximum acceleration), brake pedal is pressed when the value is between 0 and -1 (maximum braking). 0 means no pedal is pressed.
    ■ SteeringWheelAngle = Steering wheel angle (in degrees)
    ■ VehicleSpeed = Vehicle speed (in km/h)
    ■ Position X = Vehicle position along the x-axis in the simulated driving environment
    ■ Position Y = Vehicle position along the y-axis in the simulated driving environment
    ■ Position Z = Vehicle position along the z-axis in the simulated driving environment
    ■ Autonomous Mode (T/F) = Autonomous pilot status. True = autonomous pilot activated, False = autonomous pilot deactivated (driver in control of the car)

#### 2.2.3 Preprocessed data

Some of the raw collected data described above were processed and stored in the Preprocessed folder of each experiment. All experiments contain at least a Physio folder with physiological features (in a .csv file) of each participant during the different phases of the experiment (baseline and driving scenarios). Features were calculated with and without baseline correction. Also, a database containing socio-demographic information and answers to questionnaires during the experiment is located in the Questionnaire folder. Data were processed and gathered in a .csv file. A documentation file (in .xslx format) is associated to each database, containing abbreviations and item text, description, coding and range of each variable contained in the database. Besides, most of the experiments also contain Driving folder with features (reaction time, maximum steering wheel angle, ..) calculated for each takeover situation and saved in a csv file.

• /Physio: contains physiological features processed with the Neurokit library^1^ in Python [2]. Each column corresponds to a physiological indicator. More details on the significance of each indictor can be found in physiological_indicators.xlsx. Each indicator contains in its name the signal with which it has been calculated. The HRV indicators are calculated from the ECG, the RRV indicators are calculated from the RESP signal, and the RSA from the combination of the ECG and RESP signals.
  ○ /periods: contains features calculated for each period of the experiment (e.g., Baseline and Driving). The name of each file depends on the segmentation level (segm_1: features calculated on the whole periods, segm_10: signals segmented in 10 equal windows and features are calculated for each window). The baseline phase is not segmented and features are always calculated once.
  ○ /windows: contains features calculated for the driving phase, with sliding time windows with varying length and overlap. The size of time window used (60, 90 or 120 seconds) and the percentage of overlap with the previous window (0%, 25%, 50%) is specified in each file name.
  ○ Metadata:
    ■ subject_id: ID of subject
    ■ period: corresponding period of the experiment
    ■ segment_id: id of segment
    ■ time_start: time marker corresponding to the beginning of the window
    ■ time_end: time marker corresponding to the end of the window
    ■ Code for indicators: _Bl = values during baseline; _Dr = values during current period; _Dr-Bl = values during current period corrected with baseline (subtraction).
• /Questionnaire:
  ○ Exp*X*_Database.csv: contains the raw data collected in experiment *X* from the questionnaire, including socio-demographic information from participants.
  ○ Exp*X*_Documentation.xslx: contains a complete documentation for the data contained in the database. It includes terms and abbreviations, the participants to exclude for a statistical analysis (with the reason), and both the data and metadata variables (with variable name, type, description, range and coding)

### 2.3 Specificities of files folders for each experiment

In this section, the additional files or folders that are specific to each experiment are described below. Specific metadata (labels for timestamps markers, events in the driving data, ...) to each experiment are also specified here, but can also be found in the README corresponding to each experiment.

- *Exp1*
  • /Raw
    • /Physio: Metadata for labels of timestamps corresponding to experiment phases: Anfang = Start; Ende = End; Fragebogen1 = Questionnaire before the experiment; Hörbuch/Entspannung = Relaxation/Audiobook; Fragebogen2 = Questionnaire after the relaxation/audiobook phase; Probefahrt = Training phase; Fahrt = Main driving session.
    • /Driving: There is one folder for each driver, containing one .txt file for each lap (4 laps in total).
• /Preprocessed
  • /Physio: Metadata for labels corresponding to the experimental manipulation of the driver's state:
    ■ label_relaxation: 0 = No relaxation (audiobook), 1 = relaxation
    ■ label_passenger: 0 = No passenger while driving, 1 = passenger while driving
- *Exp2*

order_obstacles.csv: Order of obstacles apparition for each participant. A=Deer, B=Traffic Cone, C=Frog, D=Can, E=False Alarm1, F=False Alarm2.

• /Raw
  • /Physio: Metadata for labels of timestamps corresponding to experiment phases: Training1 = Baseline phase; Training2 = Practice phase in the driving simulator; Driving = Main driving session in conditionally automated driving.
  • /Driving:
    ■ There is one file for each driver, identified by the code of the participant.
    ■ /!\ **Due to recording problem, the “AcceleratorPedalPos” and “DeceleratorPedalPos” columns do not correspond to the gas and brake pedal position.**
    ■ /Audio: audio recording of each participant in the experimental group (in .wav format). Can be used to control for the engagement in the non-driving-related task.
• /Preprocessed:
  • /Physio: Metadata for labels corresponding to the experimental manipulation of the driver's state:
    ■ label_st: 0 = NST, not engaged in the cognitive non-driving-related task (only monitoring the environment), 1 = ST, engaged in the cognitive non-driving-related task (backward counting)
  • /Physio and Driving:
    ■ timestamps_obstacles.csv: Time elapsed (in seconds) between the start of the main driving session and the appearance of the obstacles (TrigObsX), the time when the driver pressed the button to report having understood the situation (DetObsX), and the time when the driver actually took over control (RepObsX). X corresponds to one of obstacle or the false alarm.
    ■ /takeover_epochs: features calculated for time windows shorter than 10 seconds..
      • features_tor_1s_8s_with_driving_features.csv: physiological and driving features, calculated from the signals collected from 8 seconds before to 1 second after each takeover situation.
      • features_tor_1s_8s_with_driving_features_processed.csv: Same than above but in this file, features corresponding to one driver are on the same row.
    ■ /takeover_interval: features calculated for time windows larger than 10 seconds.
      • features_tor_120s_0s.csv: physiological features calculated from the signals collected 120 seconds before each takeover situation
      • features_tor_120s_0s_processed.csv: Same than above but in this file, features corresponding to one driver are on the same row.
- *Exp3*

order_obstacles.csv: Order of obstacles apparition for each participant. A=Deer, B=Traffic Cone, C=Frog, D=Can, E=False Alarm1, F=False Alarm2. See the experimental design for further details.

• /Raw
  • /Physio: Metadata for labels of timestamps corresponding to experiment phases: Baseline = Baseline phase; Training = Practice phase in the driving simulator; BlockX = One block of the main driving session in conditionally automated driving (1 to 5). ST = Secondary task, beginning or end of a task sequence.
  • /Driving: There are three files for each driver, identified by the code of the participant: one for the baseline (<code>_Baseline.txt), one for the first two blocks (<code>_Part1.txt), and one for the last three blocks (<code>_Part2.txt).
• /Tablet: contains raw data recorded by the tablet
  • raw_data_pvt.csv: data of task performance
  • raw_data_sart.csv: data of situation awareness (SART [3] ratings and identification rate of the cause of takeover) collected after each takeover situation.
• /Preprocessed
  • /Physio
    ■ /st: contains features calculated based on signals collected during task sequences.
    ■ /periods: contains features calculated based on signals collected during each period of the experiment (Block 1 to 5).
    ■ Metadata for labels and measures corresponding to the experimental manipulation of the driver's state:
      • label_instructions: 0 = No instructions about limitations of automated vehicles before the experiment (NL), 1 = instructions received (L)
      • label_app: 0 = No context-related information through mobile application during the drive (NA), 1 = received information through app (A)
      • task_id: id of task sequence (0 to 14)
      • label_difficulty_st: 0 = No task (low), 1 = 1-back task (medium), 2 = 3-back task (high) (possibility to remove the 'No Task' condition to classifiy with two tasks)
      • label_modality_st: 0 = No task (low), 1 = visual task, 2 = auditory task (high) (possibility to remove the 'No Task' condition to classifiy with two modalities)
      • task_perf: aggregated score of task performance for this sequence, according to this formula: TaskScore = (TotalAnswers−WrongAnswers−MissedTargets)/TotalAnswers
      • nasa_score: subjective ratings of mental workload made after the task sequence (Mental Demand item of the NASA-TLX [4] questionnaire, on a 0-20 scale)
  • /Driving: contains takeover quality metrics computed during each takeover situation of the experiment for each participant
    ■ takeover_features.csv: contains the raw data
    ■ Exp3_Documentation_Takeover_Features.csv: contains the documentation of the takeover features database
- *Exp4*
• /Raw
  • /Physio: Metadata for the experiment phases: Baseline = Baseline phase; Training = Practice phase in the driving simulator; BlockX = One block of the main driving session in conditionally automated driving (1 to 2).
  • /Driving: There are three files for each driver, identified by the code of the participant: one for the baseline and training phase (<code>_Training.txt), and one each driving scenario (<code>_City/Country.txt). City = Urban area, Country = Rural area.
  • /Questionnaire: contains raw exports of the participants' answers to questionnaires, with one file for each language (German and French) in CSV format.
  • /Sleep: contains the file used by experimenter to report the information collected by the sleep tracker. They were retrieved from the desktop Fitbit application (Windows) after synchronizing the watch.
• /Preprocessed
  • /Physio
    ■ For the /periods and /windows folders, the physiological signals considered for the calculation of features are those collected during each scenario (both Rural and Urban environments), before the take-over request occurred.
    ■ /takeover_interval: features calculated for time windows larger than 10 seconds. Each file is identified by the time considered before and after the takeover request (e.g., features_tor_<time_before>_<time_after>.csv)
    ■ Metadata for labels corresponding to the experimental manipulation of the driver's state:
      • label_sleep: 0 = Not sleep deprived (A = Alert), 1 = sleep deprived (D = Drowsy)
      • label_first_scenario: Countryside (C; rural area) or Urban (U; urban area)
      • label_time_exp: 10 = 10:00am, 16 = 4:00pm
  • /Driving: contains takeover metrics for the takeover situation in each scenario. The features calculated for time windows larger than 10 seconds. Each file is identified by the time considered before and after the takeover request (e.g., features_tor_<time_before>_<time_after>.csv)
  • /PVT: contains CSV files with the participants' reaction time to targets on the psychomotor vigilance task (PVT). Participants had to press a steering wheel button when a red circle was displayed on the screen (every 5 minutes).
    ■ data_PVT_exp4_scenario_type.csv: raw values of reaction time extracted from driving data (Events column), for both environments and for each participant.
    ■ data_PVT_no_outliers_mean_sd.csv: processed values of reaction time where outliers were removed according to the mean and standard deviation of the data distribution (Threshold = Mean +/- 2*SD) [5].
    ■ data_PVT_no_outliers_quantile.csv: processed values of reaction time where outliers were removed according to the 0.05 sample quantile (Lower threshold = q0.05, higher threshold = q0.95) [5].
- *ExpTOR*
• /Raw
  • /Physio: Metadata for labels of timestamps corresponding to experiment phases:
    ■ Baseline = Baseline phase; Training = Practice phase in the driving simulator; LapX = One lap of the main driving session in conditionally automated driving (1 to 3).
  • /Driving: There are two files for each driver, identified by the code of the participant: one for the baseline and training phase (<code>-B.txt), and one the main driving session (<code>.txt).
• /Preprocessed
  • /Physio: contains features calculated from the last 90 seconds before each takeover request (TOR), for each participant and each situation.
    ■ Metadata:
      • label_environment: 0 = Adverse weather (Rainy), 1 = Nice weather (Sunny)
      • tor_modality: modality of takeover request (TOR). Ta = visual-auditory, Th = visual-haptic, Tall = visual-auditory-haptic
      • lap: in which lap the takeover was performed (out of 3)
      • reaction_time: Time elapsed in seconds between the takeover request (TOR ZONE in the raw driving data) and actual take over by the driver (Autonomous Mode (T/F) to False in the raw driving data)
      • max_swa: Maximum steering wheel angle between the takeover request and the reactivation of the autopilot
- *ExpFinal*
  • /Raw
    ○ /Driving: There are two files for each driver, one for each driving scenario (<code>_RURAL/URBAN.txt) identified by the code of the participant.
    ○ /Questionnaire: contains raw export of the participants' answers to questionnaires in CSV format.
    ○ /Limitations: contains the raw file (.xslx) with the experimenters' notes about participants' French verbal statements when a limitation was identified. It also comments about potential problems during the experiment. The file was also converted in CSV format.
    ○ /NDRT: contains raw data recorded by the tablet regarding the performance on the non-driving-related task (NDRT)
  • /Preprocessed: contains preprocessed data.
  • /AdaptiveModel: contains all the data collected by the model and logs of predictions/choices made by each module. Each subfolder contains data and logs for one module.
    ■ /driver_state: contains data collected and predictions made by the Driver State module. There are two folders for each driver, one for each driving scenario (<code>_RURAL/URBAN).
      • baseline.csv: physiological features processed in real-time during the first 90 seconds of the driving scenario, with the Neurokit library in Python [2]. They are considered as the baseline features and used for the prediction of the Driver State module.
      • features.csv: physiological features processed in real-time during the experiment with the Neurokit library in Python [2]. This was done every time new raw physiological values were collected by the sensors. Each column corresponds to a physiological indicator.
      • features_live_dr.csv: the last physiological features calculated, based on raw values of the last 90 seconds
      • features_live_all.csv: the last physiological features calculated, based on raw values of the last 90 seconds, with additional features (correction with baseline)
      • values.pkl: raw physiological values (ECG, EDA, and RESP) in the last 90 seconds of the participant
      • fusion.csv: continuous predictions made by the Driver State module every second
      • last_driver_state.pkl: array with last predicted of driver's mental workload (m2) and global driver's state (global_scale)
    ■ /supervision: contains data collected and choices made by the Supervision module for conveying information to the driver via in-vehicle interfaces. There are two files for each driver, one for each driving scenario (<code>_RURAL/URBAN.log). The lines to check in the log is the one with the "Supervision model result".
    ■ /intervention: contains predictions made by the Intervention module. There are two folders for each driver, one for each driving scenario (<code>_RURAL/URBAN).
      • tor_modality_log.csv: contains the timestamp and the prediction made by the Intervention module for the modality of take over request (TOR). 0 = visual-auditory, 1 = visual-haptic, 2 = visual-auditory-haptic.
      • last_modality.pkl: the last TOR modality predicted by the module. The value is read when the severity in the environment equals 3 (high severity), and the according modality is triggered for the TOR.
  • /Driving: contains takeover quality features for the takeover situations of the experiment. Each column corresponds to a takeover quality metric in one of the scenario (RURAL or URBAN)
  • /NDRT: contains processed data on task performance in each scenario (Rural or Urban), based on raw collected data with the tablet.
  • /Limitations: contains processed data from participants' statements about potential limitations (i.e., factors that may limit the proper functioning of the vehicle). The type, severity, and location of each limitation verbally announced by the participants were coded by two experimenters, based on the raw responses during the experiment. A documentation of the variables' name is available in this folder.

## 3 Experimental Design, Materials and Methods

The experimental design, materials, and methods used is described for each experiment separately. This information can also be found in the related published scientific papers, and in the README of each experiment.

For the driving simulation, 2 different driving simulators and 2 different open source driving simulation software were used. They are described below and referred in each experiment (Simulator and Software 1 or 2). Also, 2 different hardware were used for the collection of physiological signals. They are described below and referred in each experiment (Hardware 1 or 2).

### 3.1 Driving simulators

1. Simulator 1: Fixed-base simulator with two adjacent car seats, a steering wheel (Logitech G27), and pedals (gas and brake), as shown in Fig. 8. The driving simulation was back-projected using a projector (Epson EH-TW3200). Two speakers located behind the seats played the sound of the driving simulation to immerse drivers in the driving environment.

**Fig. 8:**
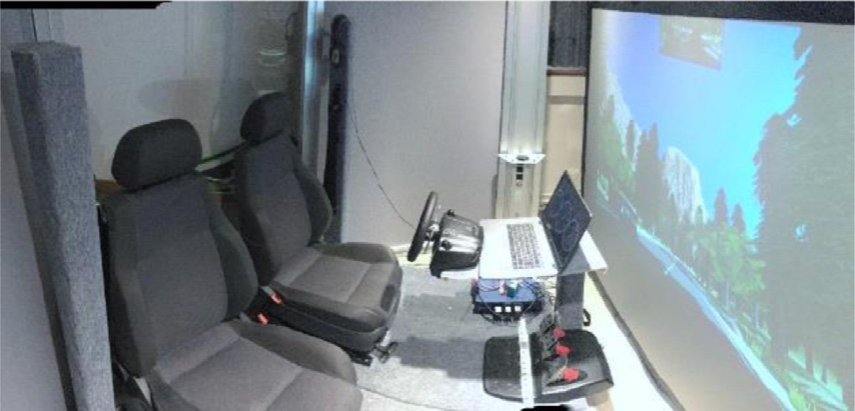
The driving simulator 1.
2. Simulator 2: Fixed-base simulator with one car seat, a steering wheel (Logitech G29), and pedals (gas and brake), as shown in Fig. 9. The driving simulation was displayed on a television screen (65").

**Fig. 9:**
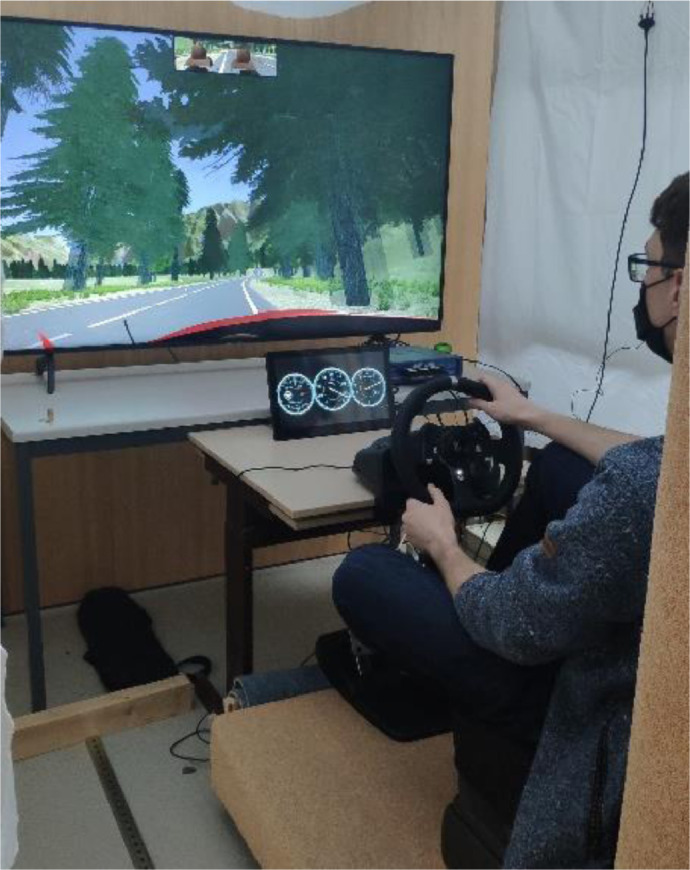
The driving simulator 2.

### 3.2 Software used for driving simulation

1. Software 1: Free version of OpenDS.
2. Software 2: GENIVI vehicle simulator^3^. The driving scenes (Yosemite, rural area; San Francisco, urban area) were modified for each experiment to match the experimental design (takeover requests and limitations in specific locations).

### 3.3 Hardware for collection of physiological signals

1. Hardware 1: BioPac Student Lab 3.7.7 software and the BioPac MP36 hardware at a sample rate of 1000 Hz. Lead sets (SS57LA and SS2LB, Biopac) with disposable Ag/AgCl pre-gelled electrodes (EL507 and EL503, Biopac) were, respectively, used to record the EDA and ECG of participants. Electrodes recording the EDA signal were placed on the distal phalanges of the middle and ring fingers of the non-dominant hand of participants. The SS5LB respiratory effort transducer (Biopac) recorded the respiration via chest expansion and contraction.
2. Hardware 2: Biosignalsplux hardware at a sample rate of 1000 Hz while running the model in Python. Lead sets with disposable Ag/AgCl pre-gelled electrodes were used to record the EDA and ECG of participants. Electrodes recording the EDA signal were placed on the distal phalanges of the middle fingers of the left hand of participants. A respiratory effort transducer recorded the respiration via chest expansion and contraction. This hardware allowed to get raw physiological values in real-time through Bluetooth collection, for processing the signals and perform the driver's state prediction continuously while driving.

### 3.4 Description of experimental design, material and methods used in each experiment

- *Exp1*
• Description of experiment: The main manipulation was to induce (social) stress by the presence of a passenger unknown to the participant. To reduce the potential negative effect of such stressor, half of drivers listened to a guided mindfulness meditation podcast for 10 minutes, while the other half (the control group) listened to an audio book (Sherlock Holmes - The Three Students). Before that, all participants listened to the audiobook for 5 minutes, as a baseline phase. Then, they had to drive for 10 minutes in the simulator. The scenario consisted of a 2 × 2 lane highway without traffic, with repeatedly occurring construction zones on the right lane. The experiment was conducted in German. More details on the experimental design and procedure, and material and instruments used can be found in [6].
• Experimental design: 2 Independent Variables (2 × 2 between-subjects design):
  ○ Between-subjects factor(s):
    ■ Presence of passenger while driving for half of participants (label_passenger)
    ■ Practice of pre-driving relaxation (listening to a guided mindfulness meditation) by half of participants (label_relaxation)
  ○ Within-subjects factor(s): None
• Experimental procedure:
  ○ 1st questionnaire > label = Fragebogen 1
  ○ Baseline (5 minutes): listening to an audiobook > label = Baseline
  ○ Audiobook/Relaxation (10 minutes): keep listening to the audiobook or listen to a guided mindfulness-meditation podcast > label = Hörbuch/Entspannung
  ○ 2nd questionnaire > label = Fragebogen 2
  ○ Training session for driving > label = Probefahrt
  ○ Driving (4 laps, 10 minutes): Manual driving on a highway without traffic > label = Fahren
• Material and instruments:
  ○ Physiological signals: Hardware 1
  ○ Driving simulation: Simulator 1 and software 1
  ○ Questionnaire: German version of the Positive and Negative Affect Schedule (PANAS) [7] (https://zis.gesis.org/skala/Breyer-Bluemke-Deutsche-Version-der-Positive-and-Negative-Affect-Schedule-PANAS-(GESIS-Panel))
- *Exp2*
• Description of experiment: The main manipulation was to induce cognitive workload to half of the participants through a verbal cognitive workload (backward counting from 3645 by steps of 2) while driving in conditional automation for 20 minutes. The other half of the participants only had to monitor the driving environment. During the driving phase, all participants had to react to 6 takeover situations, randomly triggered by the experimenter (between 1min30s and 4min after the previous one). 4 were caused by obstacles on the road (deer and frog crossing the road, traffic cone and can standing in the middle of the road) and 2 were false alarms (no obstacle on the road). The apparition order of obstacles was controlled between participants using a Latin Square design [8]. After each takeover request, participants were asked to press a button on the steering wheel once they saw and understood the situation. Then, they could choose to take over control or not, according to their evaluation of the situation being dangerous or not. They could take over control by braking, turning the steering wheel, or pressing a button on the steering wheel. Once they estimated that the situation was safe again, they were asked to reactivate the autopilot. The experiment was carried out in French, German and Italian. More details on the experimental design and procedure, and material and instruments used can be found in [9,10].
• Experimental design: 3 Independent Variables (2 × 3 × 2 mixed design):
  ■ Between-subjects factor(s):
    • Performance of verbal cognitive non-driving-related task (backwards counting) for half of participants: label_st
  ■ Within-subjects factor(s):
    • Movement of obstacle causing the takeover request: moving vs. static vs. none
    • Danger/Hazard of obstacle (i.e., potential for causing damages to the driver and the car) causing the takeover request: dangerous vs. non-dangerous vs. none
• Experimental procedure:
  ■ Baseline (5 minutes): Conditionally automated driving, driver monitors the environment > label = Baseline
  ■ Practice session (5 minutes): 3 fake takeover requests (audio-visual TOR; no obstacle on the road) + manual driving until the end of the 5 minutes > label = Training
  ■ Driving session (20 minutes): Conditionally automated driving in a rural environment without traffic > label = Driving. 6 takeover request due to obstacles: Deer, Traffic cone, Frog, Can, 2 false alarms.
• Material and instruments:
  ■ Physiological signals: Hardware 1
  ■ Driving simulation: Simulator 1 and software 2
• Questionnaires:
  • NASA Task Load Index (NASA-TLX) [4] to control for mental workload inducement
  • Situation Awareness Rating Technique (SART) [3] measured for the takeover situations (4 obstacles and once for both false alarms)
• Changes to questionnaires:
  ■ SART only with 9 items (Information quality item is missing)
  ■ Inversion of scale for 1 item of NASA-TLX for the first 29 participants. And modification of scale from 20 to 10 to make sure participants could see the whole scale without scrolling.
  ■ Questionnaires translated in French and German
- *Exp3*
• Description of experiment: Half of participants first took knowledge of limitations of automated vehicles through printed material. Then, all the participants had to perform the different N-back task sequences while the car was driving in conditional automation. The main driving session was divided in 5 blocks of 12 minutes. Each participant had to perform 3 task sequences in each block (15 task sequences in total), lasting 90 seconds each, followed by 60 seconds of rest. Participants had to rate their level of mental workload after each task sequence. In each block, a takeover occurred because of a factor limiting the operation of the automated vehicle. The N-back task type was randomized, except before the takeover request for which it was controlled with a Latin Square design [8]. After each takeover situation, participants had to rate their situation awareness and find the origin of the takeover request sent by the vehicle. Half of the participants could use an additional mobile application conveying information on the driving environment while performing the task on the tablet. These participants had to rate their user experience with this mobile application at the end of the experiment. Besides, all participants also rated their trust towards automated vehicles, both before and after the driving session. They also rated their user experience in the simulator. More details on the experimental design and procedure, and material and instruments used can be found in [11,12].
• Experimental design: 4 Independent Variables (2 × 2 × 3 × 3 mixed design)
  ■ Between-subjects factor(s):
    • Presentation of automated vehicles limitations: label_instructions
    • Use of an additional mobile application to receive context-related information on the driving environment: label_app
  ■ Within-subjects factor(s):
    • Task difficulty (no task vs. 1-back vs. 3-back): label_difficulty_st
    • Task modality (no task vs. visual vs. auditory): label_modality_st
• Experimental procedure:
  ■ Baseline (5 minutes): Conditionally automated driving, driver monitors the environment > label = Baseline
  ■ Training session (5 minutes): 3 fake takeover requests (audio-visual TOR; no obstacle on the road) + manual driving until the end of the 5 minutes > label = Training
  ■ Driving session (around 1 hour): Conditionally automated driving in a rural environment without traffic
    • 5 blocks and 1 takeover request per block > label = Block1, Block2 ... Block5
    • 3 sequences of non-driving-related task per block > label = task_id (0 to 14)
    • IDs of task sequences in which a takeover occurred: 2 (Slope), 4 (Lanes), 7 (Rock), 9 (Rain), 13 (Deer)
• Material and instruments:
  ■ Physiological signals: Hardware 1
  ■ Driving simulation: Simulator 1 and software 2
• Questionnaires:
  ■ Mental Demand item of the NASA Task Load Index (NASA-TLX) [4] to control for mental workload inducement
  ■ Situation Awareness Rating Technique (SART) [3] (collected after each takeover situation)
  ■ Scale of Trust in Automated Systems [13]
  ■ Official French and German versions of the User Experience Questionnaire Short version (UEQ-S) [14], used to measure user experience in the driving simulator and with the mobile application (half of participants)
• Changes to questionnaires:
  ■ Trust in automated system questionnaire was changed to "trust in automated driving systems"
  ■ Trust questionnaire, SART and NASA-TLX were translated to French, German and Italian due to no official validated translation
  ■ Using only the *Mental Demand* item from the NASA-TLX questionnaire to control for mental workload inducement
- *Exp4*
• Description of experiment: All participants were asked to come the day before the experiment to collect a sleep tracker (smart watch) and to be given instructions about their sleep (sleep deprived or not). The time of the experiment (10am or 4pm) was controlled to ensure that it did not impact alertness levels. On the day of the experiment, participants first rated their level of fatigue and affective state (valence and arousal). Then they observed the car driving autonomously for 5 minutes, which was considered the baseline phase for physiological measures. Afterwards, the participants were instructed to test the simulator and learn about the principle of takeover request (TOR). Then, the main driving session consisted of two 30-minute scenarios in each of the two environments (urban or rural). The order of the scenarios was controlled: half of the participants started with the rural, and the other half with the urban. Drivers were required to observe the environment, so that the task was monotonous and an increase in drowsiness could be observed. They also had to press a button on the steering wheel when a target (red circle) appeared on the screen every 5 minutes (vigilance task). After each scenario, participants rated their fatigue (before the takeover and after answering all the questions), their emotional state, their situational awareness at the time of the TOR, and their confidence in the car. Finally, they rated their user experience in the simulator. The experiment was conducted in French and German.
• Experimental design: 4 Independent Variables (2 × 2 × 2 × 2 mixed design):
  ○ Between-subjects factor(s):
    ■ Sleep deprivation: less than six hours of sleep the night before the experiment vs. more than seven hours: label_sleep
    ■ Scenario order: driving in rural area first vs. driving in urban area first: label_first_scenario
    ■ Time of experiment: 10:00am vs. 4:00pm: label_time_exp
  ○ Within-subjects factor(s):
    ■ Driving environment: Rural area vs. urban area: period
• Experimental procedure:
  ○ Baseline (5 minutes): Conditionally automated driving, driver monitors the environment > label = Baseline
  ○ Training session (5 minutes): 3 fake takeover requests (audio-visual TOR; no obstacle on the road) + manual driving until the end of the 5 minutes > label = Training
  ○ Driving session (around 1 hour): Conditionally automated driving in 2 scenarios: A rural environment and an urban one > label = Block1, Block2
• Material and instruments:
  ○ Physiological signals: Hardware 1
  ○ Driving simulation: Simulator 2 and software 2
• Questionnaires:
  ○ Karolinska Sleepiness Scale (KSS) [15] to measure self-reported fatigue
  ○ Animated Self Assessment Manikin (AniSAM) [16] to assess the drivers' affective state (valence and arousal)
  ○ Situation Awareness Rating Technique (SART) [3] to measure the drivers' situation awareness in both takeover situations
  ○ The Situational Trust Scale for Automated Driving (STS-AD) [17], to measure trust in the vehicle in both environments
  ○ Official French and German versions of the User Experience Questionnaire Short version (UEQ-S) [14], to measure user experience in the driving simulator
• Changes to questionnaires:
  ○ Questionnaires were translated in French and German when no official translation could be found
- *ExpTOR*
• Description of experiment: Participants started the experiment by sitting in the simulator and monitoring the car's environment while it was driving autonomously for 5 minutes. This was used as the baseline measure for physiological data. Afterwards, the participants were instructed to test the simulator and learn about the principle of takeover request (TOR). Then, the main driving session consisted of three laps lasting 12 minutes each in a rural environment without traffic. In each lap, drivers were required to engage in a different NDRT (visual 2-back task vs. auditory 2-back task vs. no task) and take over control of the car accordingly when requested. They performed the task on a handheld device. Besides, they had to take over control three times in each lap, with each takeover request through a different modality: icon on the dashboard and audio chime (audio-visual), icon on the dashboard and vibrations in the seat (audio-haptic), or a combination of all three (audio-visual-haptic). In total, the participants encountered 9 takeover situations each, caused by a fixed obstacle appearing on a road with a time-to-collision of around 7 seconds. For half of participants, the weather was always sunny, whereas it was rainy for the other half. The experiment was conducted in French. More details on the experimental design and procedure, and material and instruments used can be found in [18].
• Experimental design: 3 Independent Variables (2 × 3 × 3 mixed design):
  ○ Between-subjects factor(s):
    ■ Weather condition: sunny (S) vs. rainy (R)
  ○ Within-subjects factor(s):
    ■ Non-driving-related task (NDRT): visual 2-back task vs. auditory 2-back task vs. no task
    ■ Takeover modality: visual-auditory vs. visual-haptic vs visual-auditory-haptic
• Experimental procedure:
  ○ Baseline (5 minutes): Conditionally automated driving, driver monitors the environment > label = Baseline
  ○ Training session (3 minutes): 3 fake takeover requests (TORs, 1 of each modality) + manual driving until the end of the 5 minutes > label = Training
  ○ Driving session (around 36 minutes): 3 laps of conditionally automated driving, with 1 NDRT performed in each lap > label = Lap1, Lap2, Lap3
• Material and instruments:
  ○ Physiological signals: Hardware 1
  ○ Driving simulation: Simulator 2 and software 2
• Questionnaire:
  ○ Official French version of the User Experience Questionnaire Short version (UEQ-S) [14], to measure user experience in the driving simulator
- *ExpFinal*
• Description of experiment: On the day of the experiment, participants first rated their level of fatigue and affective state (valence and arousal). The participants started the experience in the driving simulator with a training session to become familiar with the driving controls and the takeover request (TOR) principle. Then, the main driving session consisted of two 10-minute scenarios in two environments (first rural then urban area). Each scenario started with a period of 90 seconds while participants only had to monitor the vehicle's environment and no takeover could be requested. This phase was used to calculate the baseline physiological features of drivers, used afterwards by the model. During each scenario, participants had to engage in a cognitive NDRT (visual 2-back task) on a handheld device at certain moments. Otherwise, they were asked to monitor the vehicle's environment. Half of participants received additional context-related information through in-car interfaces (Supervision module): ambient lights on the dashboard showing global severity of the environment, vibration in the seat to warn about lane markings state and obstacles, and pop-up icons on the handheld device with the severity and type of limitation. The other half did not receive any additional information. Besides, the takeover request modality was smartly selected (visual-auditory, visual-haptic, or visual-auditory-haptic) for half of participants (Intervention module), depending on their current physiological state (last 90 seconds). The other half were required to take over with a unique visual-auditory modality. All drivers had to take over control once in each scenario. Besides, the Driver State module continuously predicted the driver's state (every second) using the physiological features of the last 90 seconds, according to four components: fatigue, mental workload, affective state and situation awareness. At the end of each scenario, drivers had to rate their situation awareness, mental workload, situational trust towards the vehicle, affective state, and fatigue. at the end of the experiment, they were asked to rate their user experience in the simulator, as well as giving feedbacks. The experiment was conducted in French. More details on the experimental design and procedure, and material and instruments used can be found in [19].
• Experimental design: 3 Independent Variables (2 × 2 × 2 mixed design)
  ○ Between-subjects factor(s):
    ■ Supervision: availability of the Supervision module vs. not
    ■ Intervention: availability of the Intervention module vs. no
  ○ Within-subjects factor(s):
    ■ Driving environment: Rural area vs. urban area
• Experimental procedure:
  ○ Training session (5 minutes): Explanation of Supervision/Intervention modules if available + manual driving until the end of the 5 minutes
  ○ Driving session (around 20 minutes): Conditionally automated driving in 2 scenarios. A rural environment and an urban one > label = Rural, Urban. Each scenario started with a baseline of 90 seconds while the car was driving (getting baseline physiological features)
    ■ Scenario 1: Rural area
    ■ Scenario 2: Urban area
• Material and instruments:
  ○ Physiological signals: Hardware 2
  ○ Driving simulation: Simulator 2 and software 2
• Questionnaires:
  ○ Mental Demand item of the NASA Task Load Index (NASA-TLX) [4] to get self-reported mental workload during each scenario
  ○ Karolinska Sleepiness Scale (KSS) [15] to measure self-reported fatigue
  ○ Animated Self Assessment Manikin (AniSAM) [16] to assess the drivers' affective state (valence and arousal)
  ○ Situation Awareness Rating Technique (SART) [3] to measure the drivers' situation awareness in both takeover situations
  ○ The Situational Trust Scale for Automated Driving (STS-AD) [17], to measure trust in the vehicle in both environments
  ○ User Experience Questionnaire Short version (UEQ-S) [14], to measure user experience in the driving simulator
• Changes to questionnaires:
  ○ Translate in French when no official translation existed

## Ethics statements

We confirm that relevant informed consent was obtained from all subjects in the six experiments carried out. The research was carried out in accordance with the Declaration of Helsinki, and approved by the Ethical committee of the department of Psychology (protocol number IRB-445) at the University of Fribourg (Switzerland).

## CRediT authorship contribution statement

**Quentin Meteier:** Methodology, Software, Formal analysis, Investigation, Data curation, Writing – original draft, Visualization. **Marine Capallera:** Methodology, Software, Formal analysis, Investigation. **Emmanuel de Salis:** Methodology, Software, Formal analysis, Investigation. **Leonardo Angelini:** Conceptualization, Supervision, Validation, Funding acquisition. **Stefano Carrino:** Conceptualization, Supervision, Validation, Funding acquisition. **Marino Widmer:** Supervision, Validation. **Omar Abou Khaled:** Resources, Funding acquisition. **Elena Mugellini:** Resources, Conceptualization, Supervision, Project administration, Funding acquisition. **Andreas Sonderegger:** Conceptualization, Methodology, Supervision, Validation, Formal analysis, Investigation, Resources, Funding acquisition, Writing – review & editing.

## Declaration of Competing Interest

The authors declare that they have no known competing financial interests or personal relationships that could have appeared to influence the work reported in this paper.

## Data Availability

A dataset on the physiological state and behavior of drivers in conditionally automated driving (Original data) (Zenodo).

## Footnotes

1 https://neuropsychology.github.io/NeuroKit

3. GENVI Vehicle simulator. https://github.com/GENIVI/genivi-vehicle-simulator
